# Effects of Etching Time and Ethanol Wet Bonding on Bond Strength and Metalloproteinase Activity in Radicular Dentin

**DOI:** 10.3390/jcm13092474

**Published:** 2024-04-24

**Authors:** Allegra Comba, Andrea Baldi, Riccardo Pucci, Chiara Rolando, Mario Alovisi, Damiano Pasqualini, Nicola Scotti

**Affiliations:** Department of Surgical Sciences, Dental School Lingotto, University of Turin, 10100 Turin, Italy; allegra.comba@unito.it (A.C.); andrea.baldi@unito.it (A.B.); riccardo.pucci@edu.unito.it (R.P.); chiara.rolando225@edu.unito.it (C.R.); mario.alovisi@unito.it (M.A.); damiano.pasqualini@unito.it (D.P.)

**Keywords:** endodontic treatment, fiber posts, metalloproteinases, ethanol pre-treatment, multi-mode universal adhesive, push-out test, nanoleakage, in situ zymography

## Abstract

(1) **Background**: The objective of this in vitro study was to evaluate the impact of different etching times and ethanol pre-treatments on the immediate bond strength of a hydrophilic multi-mode universal adhesive (Clearfil Universal Bond Quick, Kuraray, UBQ) and on the consequent gelatinolytic activity of metalloproteinases (MMPs) on radicular dentin. (2) **Methods**: Sixty single-root teeth were selected and divided into four groups according to the adhesive protocol applied for fiber post cementation: (G1) 15 s H_3_PO_4_ application + UBQ; (G2) 30 s H_3_PO_4_ application + UBQ; (G3) 15 s H_3_PO_4_ application + ethanol pre-treatment + UBQ; (G4) 30 s H_3_PO_4_ + ethanol pre-treatment + UBQ. After adhesive procedures, fiber posts were luted into the post space with a dual-curing cement (DC Core, Kuraray) and light-cured for 40 s. To perform the push-out test and nanoleakage analyses for both coronal end apical areas, 1 mm slices were prepared, following a 24 h storage period in artificial saliva. Additionally, an in situ zymographic assay was conducted to explore endogenous MMP activity within the radicular layer. Results were statistically analyzed with ANOVA and Tukey post hoc tests. Statistical significance was set at *p* < 0.05. (3) **Result**: ANOVA revealed a statistically significant difference in push-out bond strength related to the pre-treatment variable but did not highlight any significance of etching time. Specimens pre-treated with ethanol wet bond application showed higher bond strength (*p* < 0.01). In situ zymography quantification analyses revealed that all tested groups, independently of etching time end ethanol pre-treatment, activated MMP gelatinolytic activity. A significant increase in MMP activity was detected for the 30 s etching time. However, ETOH pre-treatment significantly reduced MMP activity within the adhesive interface (*p* < 0.01). (4) **Conclusions**: The tested adhesive showed similar results regardless of the etching time protocol. The gelatinolytic activity of MMPs was observed in all the groups. Further investigations and extended follow-ups are required to validate the results of the present study in vivo.

## 1. Introduction

Fiber posts combined with adhesively bonded materials are commonly used to restore non-vital teeth due to the structural consequences that occur after endodontic treatment [1,2,3]. The elastic modulus of fiber posts and radicular dentin is similar, and for this reason, their use for the creation of tooth restoration units with improved resistance to dislodgement [2] and reduced risk of irreparable root fracture [4] has been proposed.

Dental adhesion plays a crucial role in the effectiveness of restorations supported by fiber posts. Therefore, it is essential to select the proper bonding protocols for luting fiber posts to dentin in root canals to ensure the long-term success of post-endodontic restorations. Different methods and resin cements are employed to establish a durable and stable bond between the fiber post and the tooth structure. Surface treatments, including silanization, etching, cleaning with alcohol, or air abrasion, enhance the bond strength and long-term stability of fiberglass posts. Moreover, maintaining an ideal moisture level in the dentin during cementation is crucial for attaining dependable and enduring bonding [5]. Similar to coronal dentin, the radicular substrate can also be prepared using two methods: the etch-and-rinse (ER) method or the self-etch (SE) method [5,6,7,8].

In recent times, a new type of adhesive, known as a “universal” or “multi-mode” adhesives, has emerged. These adhesives are designed to simplify the bonding process by combining acidic priming and bonding into one solution. They can be used with various approaches, such as etch-and-rinse, selective-etch, or self-etch. A recent study conducted by Munoz et al. found that some adhesives applied in the self-etch mode on the upper part of the tooth generated a weaker bond strength in comparison to the etch-and-rinse method. However, certain adhesives were able to achieve similar bond strength regardless of the application mode [9]. Information on the bond strength of multi-mode adhesive systems when used for fiber post cementation on radicular dentin is available in the literature [10]. Data on bond strength to radicular dentin using various application methods have been reported but little knowledge is available on different times of etching application.

Moreover, it is well known that the conditioning of dentin exposes and activates endogenous matrix metalloproteinases (MMPs) which are incorporated into the mineralized dentin during the last phase of dentinogenesis [10]. Proteinases like MMP-2, MMP-8 and MMP-9 [11,12] exist in both coronal and radicular dentin [13] and play a significant role in the degradation of exposed collagen at the resin–dentin interface [14,15,16,17,18], thus causing the failure of the adhesive interface over time [19]. More specifically, the exposed collagen, located at the base of the hybrid layer, undergoes gradual destruction by proteases that are either directly or indirectly bound to the collagen fibrils. This process leads to the weakening of the anchoring function of the hybrid layer, consequently reducing bond strength at the adhesive interface [20]. In fact, the most common reason for clinical failure of endodontically treated teeth restored with post and core build-ups is fiber post debonding from radicular dentin [21].

Another crucial concern regarding the stability of the hybrid layer is the inadequate infiltration of demineralized dentin. It is essential to have water present in the etched dentin to prevent the collagen fibers from coming together, facilitating improved permeation of the adhesive monomers into the collagen framework and dentin tubules [21]. However, if there are high levels of humidity during monomeric infiltration, it can cause the hydrophilic and hydrophobic monomers to separate [20], which negatively impacts monomeric conversion [22] and results in the creation of imperfect hybrid layers [20,23]. Previous findings have found that pre-treating by applying ethanol to coronal dentin would benefit adhesion bond strength [24]. Researches have also investigated the application of ethanol to radicular dentin and demonstrated a positive effect of the procedure on post space cementation [24]. However, no previous study has specifically investigated the assessment of etching time and the use of ethanol alongside multi-mode adhesive systems in radicular dentin and its effect on MMP activity. These factors are important to consider in terms of both bond strength and preservation of the adhesive interface. Hence, the purpose of this research was to examine the impact of varying etching times and ethanol pre-treatment on [1] bond strength and [2] the activity of MMPs within the hybrid layer of a multi-mode universal adhesive system on radicular dentin. The null hypothesis tested was twofold: on the one hand, that the push-out bond strength to radicular dentin is not affected by etching time and ethanol pre-treatment, and on the other, that the gelatinolytic activity of MMPs is not influenced by the variable tested.

## 2. Materials and Methods

### 2.1. Sample Preparation

This research study was granted ethics approval by the local ethics committee of the Dental School, University of Turin (DS-001/2021).

A total of 60 mono-radicular and round-shaped single canal human teeth, extracted for periodontal or orthodontic reasons, were selected for this study (patient age range 20–40). The following selection criteria were followed: no presence of radicular caries or fractures, no history of endodontic, restorative, or prosthetic treatments. The specimens were stored in distilled water for the debridement phase and then immediately preserved in 0.5% chloramine solution at 4 °C for no longer than 3 months after extraction. Each specimen was sliced beneath the cemento-enamel junction using a diamond saw, followed by an endodontic procedure performed using rotary nickel–titanium instruments (Waveone^®^ GOLD, Dentsply Maillefer, Ballaigues, Switzerland). This was done while irrigating with 5% NaOCl (Niclor 5; Ogna, Muggiò, Italy) alternated with 2 mL 10% EDTA (Tubuliclean; Ogna, Muggiò, Italy). The working length was established to maintain a distance of 1 mm from the visible apical foramen. In accordance with the continuous wave technique, the cavity was thoroughly rinsed with water and dried using paper points. Then, specimens were filled with gutta-percha points (Gutta Percha Points Medium, Inline; B.M. DentaleSas, Turin, Italy) using a heat source (Down Pack, Hu-Friedy, Chicago, IL, USA) and an endodontic sealer (Pulp Canal Sealer EWT; Kerr, Orange, CA, USA). Backfilling was performed with heated gutta-percha (Obtura Max system, Analytic Technologies, Redmond, WA, USA).

The samples were kept in distilled water for one week. In each sample, an 8 mm post space was prepared using dedicated drills (Rebilda Post #15 VOCO, Cuxhaven, Germany). The right length of each fiber post (Rebilda Post #15 VOCO, Cuxhaven, Germany) was verified. Post spaces were then rinsed with 5 mL physiological saline solution (NaCl) to eliminate any leftover debris. Lastly, the fiber posts were cleaned with ethanol for 30 s.

Specimens were arbitrarily divided into 4 groups based on the dentin acid-etching (K-etch, Kuraray, Tokyo, Japan) treatment:

- Group 1: 15 s 36% H_3_PO_4_ application;
- Group 2: 30 s 36% H_3_PO_4_ application;
- Group 3: 15 s 36% H_3_PO_4_ application, then post spaces were filled with 100% EtOH for 1 min;
- Group 4: 30 s 36% H_3_PO_4_ application, then post spaces were filled with 100% EtOH for 1 min.

In every sample, phosphoric acid was always rinsed for 60 s with water and dried with paper points and oil-free air spray. A multi-mode adhesive system (Clearfil Universal Bond Quick, Kuraray, Tokyo, Japan) was chosen and applied to the acid-etched post space as well as each fiber post, adhering to the guidelines provided by the manufacturer. Next, dual-cure luting cement (DC Core, Kuraray, Tokyo, Japan) was prepared in line with the manufacturer’s directions and introduced into the post space via a mixing tip of the appropriate size. Fiber posts were gradually placed into the post space and excess cement was removed. Every specimen was light-cured for 2 min (30 s for each side) using an LED lamp (Valo, Ultradent, South Jordan, UT, USA).

### 2.2. Push-Out Test

After 24 h, 9 specimens from each group were utilized for the push-out test. Slices of radicular dentin 1 mm in thickness were obtained from each specimen using a low-speed diamond saw (Micromet, Remet, Casalecchio di Reno, Italy) under water cooling and stored in artificial saliva. Every segment was marked on its frontal aspect using a permanent marker. Once sectioned, specimens were stored in artificial saliva at 37 °C, prepared in accordance with the protocol of Pashley et al. (2004) [25]. From each specimen, 4 to 5 slices of the coronal/apical post space portion were obtained. Each slice’s thickness was measured with a digital caliber with 0.01 mm accuracy and was then photographed on a millimeter paper to allow for the measurement of the bonding area with ImageJ. The push-out test was conducted by applying axial pressure on the post at a crosshead velocity of 0.5 mm/min utilizing an Instrom Machine I model 10/D (Sintech, MTS, 14000 Technology Drive, Eden Prairie, MN 55344, USA). The highest coronal portion was always oriented downward (load direction: from apical to coronal). Peak failure load was noted in newtons (N) and transformed into megapascals (MPa) according to the surface adhesion area by dividing the load in newtons by the bonded surface area (*S*L) in mm^2^. SL was calculated as the lateral surface area of a truncated cone utilizing the formula: S_L_ = π (R + r) [(h^2^ + (R − r))^2^]^0.5^ π where *R* is the coronal post radius, *r* represent the apical post radius and *h* root slice thickness.

### 2.3. Nanoleakage Evaluation

For nanoleakage assessment, three samples from each group were also chosen and utilized. Specimens were sectioned into slices of 1 mm thickness, then immersed in silver nitrate solution in 50 wt% ammoniacal silver nitrate (AgNO_3_) for 24 h. They were then dipped in a photo-developing solution, affixed to glass slides and smoothed down using SiC paper under flowing water. Afterward, they were dyed with a 0.5% solution of acid fuchsin for 15 min. Observations were conducted using a light microscope (Nikon E800; Nikon, Tokyo, Japan). Images of the adhesive interfaces were obtained (original magnification: 1000×) and the degree of interfacial nanoleakage was scored on a scale of 0–4 by two observers. The reliability of the examiner was determined using the kappa (κ) test.

### 2.4. In Situ Zymography

Lastly, in situ zymography evaluation procedures were performed using the method reported by Mazzoni et al. (2012, 2014) [24,26]. Specimens were divided into the previously cited groups and, following fiber post cementation, six 1 mm thick sections, cut perpendicular to the fiber post axis, were created from each sample using a low-speed diamond saw (Micromet, Remet, Bologna, Italy) under water cooling. The sections were subsequently adhered to glass slides and then ground down to achieve a thickness of roughly 50µm for each specimen. To create the MMP substrate, a 1.0 mg/mL concentration of a stock solution made up of self-quenched fluorescein-conjugated gelatin (E-12055, Molecular Probes, Eugene, OR, USA) was produced by introducing 1.0 mL of deionized water into the vial containing the lyophilized gelatin. The MMP substrate was then kept at −20 °C until use. The gelatin stock solution was diluted 10 times with dilution buffer (NaCl 150 mM, CaCl_2_ 5 mM, Tris-HCl 50 mM, pH 8.0), followed by the introduction of an anti-fading agent (Vectashield mounting medium with 4′,6-diamidino-2-phenylindole (DAPI), Vector Laboratories Inc., Burlingame, CA, USA). Each polished dentin section was topped with fifty microliters of the fluorescent gelatin mixture and safeguarded with a cover slip. The assemblies of the glass slide were sheltered from light and incubated in a moisture-controlled chamber at a temperature of 37 °C for 12 h. The hydrolysis of quenched fluorescein-conjugated gelatin substrate within the hybrid layer, indicative of endogenous gelatinolytic enzyme activity, was evaluated through scrutinizing the glass slides under a multi-photon confocal laser scanning microscope (TCS SP5-AOBS 5-channel, Leica Microsystems - Wetzlar, Germania), using an excitation wavelength of 495 nm and an emission wavelength of 515 nm. Images were acquired using a HCX PL APO 40X 1.25 NA oil immersion objective. Optical sections (350 nm thick) were obtained from varying focal planes. ImageJ software 2.1.0/1.53c (http://imagej.nih.gov/ij/docs/index.html) was used for the analysis (access date 2 August 2022), quantification and processing of the stacked images. The emission of fluorescence intensity from the hydrolyzed fluorescein-conjugated gelatin was quantified. The volume of gelatinolytic activity was expressed as integrated dentin (Arbitrary Unit, AU) of the green fluorescence within the hybrid layer.

### 2.5. Statistical Analysis

Bond strength data, which were found to be normally distributed (Kolmogorov–Smirnov test), were processed using a three-way ANOVA. This was done to study the impacts of the variables “etching time” (15 s or 30 s), “ethanol pre-treatment” and “post space area” (coronal or apical) and their interactions on bond strength. The Tukey test was utilized for post hoc pairwise comparisons. The chi-square test was employed to analyze differences in failure modes. In situ zymography quantification was statistically evaluated with a two-way ANOVA (factors: “etching time”, “ethanol pre-treatment”) and the post hoc Tukey test. For all statistical evaluations, statistical significance was pre-set at *p* < 0.05. Stata 12.0 software (StataCorp, College Station, TX, USA) was used to carry out all the statistical analyses.

## 3. Results

### 3.1. Push-Out Bond Strength Test

Table 1 illustrates the average and standard deviation of the push-out bond strength, denoted in MPa. The three-way ANOVA revealed that significant differences were observed for the factors “etching time” (F = 23.08, *p* < 0.01), “ethanol pre-treatment” (F = 14.96, *p* < 0.01) and “post space area” (F = 26.06, *p* < 0. 01). The interactions between the factors “etching time” and “ethanol pre-treatment” (F = 4.29, *p* = 0.041) and between “etching time” and “post space area” (F = 13.92, *p* < 0.01) were also significant. However, the interaction between the factors “ethanol pre-treatment” and “post space area” was not significant (F = 0.45, *p* = 0.50). Similarly, the interaction of all three factors, “etching time”, “ethanol pre-treatment” and “post space area”, was not significant (F = 2.46, *p* = 0.12).

### 3.2. Nanoleakage Analysis

**Figure 1:**
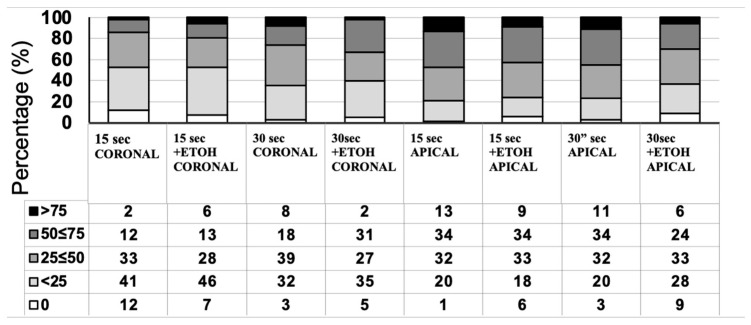
The assessment of interfacial nanoleakage was carried out in all groups, with the percentage of sections and nanoleakage ratings detailed. Interfacial nanoleakage was scored based on the percentage of the adhesive surface showing silver nitrate deposition: 0, no nanoleakage; 1, <25% with nanoleakage; 2, 25% to ≤50% with nanoleakage; 3, 50% to ≤75% with nanoleakage; 4, >75% with nanoleakage. Increasingly darker grey shades indicate increasing interfacial nanoleakage expression. N = number of analyzed sections.

**Figure 2:**
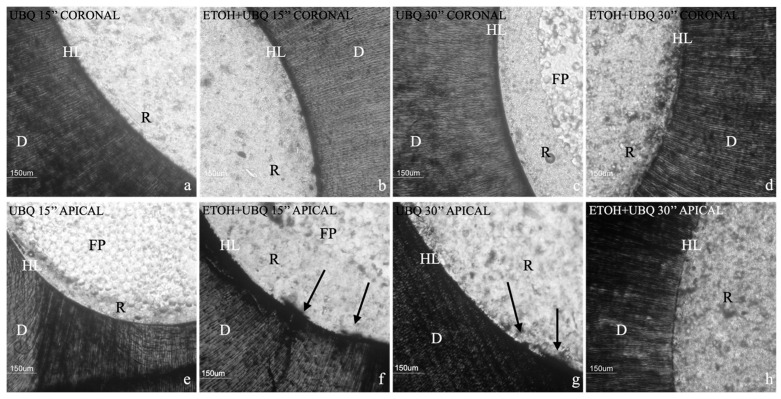
Nanoleakage analysis. R, resin cement; FP, post; D, dentin; HL, hybrid layer. (**a**) Example image of nanoleakage assessment in coronal group 1, where no leakage was detected in the observed area. (**b**) Example image of nanoleakage assessment in coronal group 2, where no leakage was detected in the observed area. (**c**) Example image of nanoleakage assessment in coronal group 3, where no leakage was detected in the observed area. (**d**) Example image of nanoleakage assessment in coronal group 4, where no leakage was detected in the observed area. (**e**) Example image of nanoleakage assessment in apical group 1, where no leakage was detected in the observed area. (**f**) Example image of nanoleakage assessment in apical group 2; leakage is indicated by black arrows in the observed area. (**g**) Example image of nanoleakage assessment in apical group 3; leakage is indicated by black arrows in the observed area. (**h**) Example image of nanoleakage assessment in apical group 4, where no leakage was detected in the observed area.

Figure 1 illustrates a descriptive statistical analysis of interfacial leakage scores. There were no statistically significant differences among the groups regarding the penetration level of silver nitrate (*p* = 0.052). However, a negative trend was visible for 15″ and 30″ etching times for the apical region, as shown in Figure 1 and Figure 2.

### 3.3. In Situ Zymography

**Figure 3:**
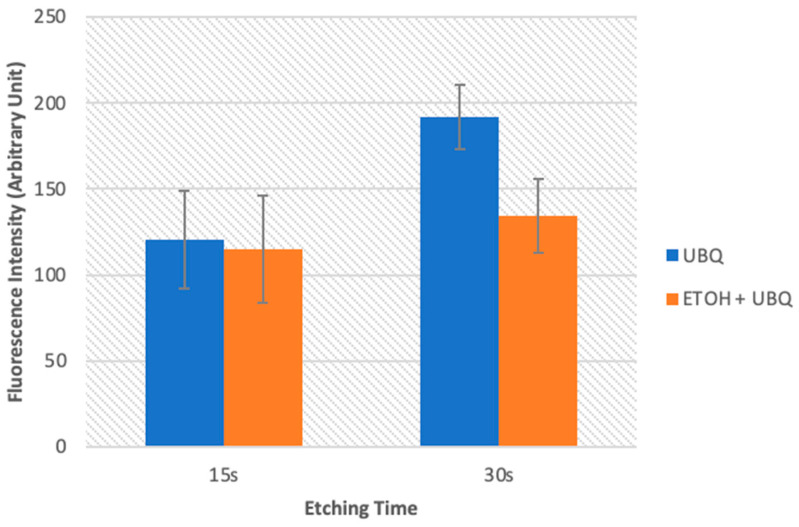
Gelatinolytic activity is represented as the proportion of green fluorescence within hybrid layers established with Universal Bond Quick (UBQ). This is either with or without preliminary treatment with carbodiimide ethanol (ETOH) following 15 s or 30 s of acid etching on post space dentin. Values are represented as means and standard deviations. For the comparison of the factor “etching time”, columns labeled with the same uppercase letters are not significantly different (*p* > 0.05). For the comparison of the factor “ETOH pre-treatment”, columns labeled with the same numerals are not significantly different (*p* > 0.05).

**Figure 4:**
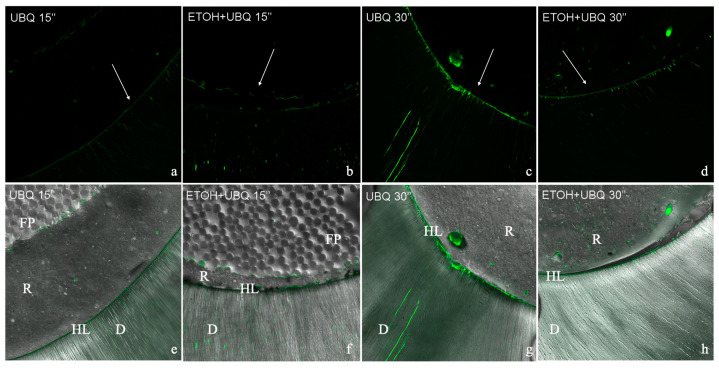
Images captured through confocal laser scanning microscopy depicting the interfaces of radicular dentin bonded with resin which were subjected to incubation with gelatin and labeled with quenched fluorescein. Green luminescence indicates regions where inherent gelatinolytic activity was high within the dentinal tubules and the hybrid layer. The image at the bottom was created by combining the differential interference contrast image (showing the optical density of the resin–dentin interface) and the image captured in the green channel. HL, hybrid layer; D, dentin; FP fiber post; R, resin cement. (**a**) Example darkfield image of the green fluorescence detected at the adhesive interface in group 1. (**b**) Example darkfield image of the green fluorescence detected at the adhesive interface in group 2. (**c**) Example darkfield image of the green fluorescence detected at the adhesive interface in group 3. (**d**) Example darkfield image of the green fluorescence detected at the adhesive interface in group 4. (**e**) Example brightfield image of green fluorescence combined with a differential interference contrast image for group 1. (**f**) Example brightfield image of green fluorescence combined with a differential interference contrast image for group 2. (**g**). Example brightfield image of green fluorescence combined with a differential interference contrast image for group 3. (**h**) Example brightfield image of green fluorescence combined with a differential interference contrast image for group 4.

Figure 3 illustrates the level of gelatinolytic activity represented as integrated dentin (Arbitrary Unit, AU) of the green fluorescence found within the hybrid layer. In all the specimens tested, in situ zymography showed an intense green fluorescence in the mineralized radicular dentin within the hybrid layer, suggesting that the fluorescein-conjugated gelatin was significantly hydrolyzed at these sites (Figure 2 and Figure 4). Two-way ANOVA demonstrated that the activation of MMP gelatinolytic activity was consistent across all examined groups, independently of etching time and ethanol pre-treatment. A significant increase in the activity of MMPs was observed for the 30 s etching time. However, pre-treatment with ETOH significantly decreased MMP activity within the adhesive interface (*p* < 0.01).

## 4. Discussion

Considering the results obtained in the present in vitro study, the initial null hypothesis was rejected, since bond strength was significantly influenced either by the etching time or by the ethanol pre-treatment.

Theoretically, the more the dentin is etched, the more the adhesive penetrates the demineralized substrate. On the other hand, the adhesive’s penetration into demineralized dentin often leads to deficient resin infiltration due to moisture inhibition at the hybrid bottom layer containing denuded collagen fibrils [27,28]. Independently of dentin pre-treatment, the present study showed that etching time was significantly correlated with fiber post bond strength to radicular dentin. In radicular dentin, especially after post space preparation, the created smear layer is quite thick [29] and large areas of dentin covered with gutta-percha and sealer remnants have been detected [30]. Prolonged etching time could lead to a more complete removal of either the debris produced during post space preparation or the peritubular dentin nearest to the surface, thus increasing the wettability of radicular dentin, which leads to higher resin infiltration [31]. On the other hand, research conducted on coronal dentin has shown that when phosphoric acid is applied for longer periods of time, it leads to increased exposure of demineralized collagen fibril. However, this extended application time is associated with lower biochemical preservation of collagen and proteoglycans in the dentin matrix [32]. This is probably correlated with acid-induced structural modifications occurring when 35% phosphoric acid remains in contact with denuded collagen for more than 15 s [32]. However, in radicular dentin, there is no need to worry about a potential risk of post-operative sensitivity being caused by dentin over-etching and insufficient demineralized dentin infiltration [33]. An increased phosphoric acid application up to 30 s led to a significant increase in bond strength when a mild acidic universal adhesive was employed, thus suggesting that it could be a suitable bonding technique for fiber post luting.

Furthermore, a previous study showed that various regions of an identical root canal did not react equally to the acid etching procedure [34]. Adhesive systems are less likely to effectively infiltrate apical root dentin because of reduced tubule density, cementum-like tissue, irregular secondary dentin and numerous accessory canals [35]. Previous studies using SEM indicated that the process of adhesive bonding to root dentin also hinges on the generation of resin tags [36]. According to Gwinnett [37], these tags are responsible for approximately 30% of overall bond strength. Even with a universal adhesive, applied in an etch-and-rinse mode, the present finding confirms a reduced bond strength to the apical post space portion, independently of phosphoric acid application time. The highest bond strength to the post space coronal region is attributed to the greatest amount of moisture control and the most accessible part of the canal space [34] during etching and applying the adhesive agent [38]. Furthermore, it was emphasized that there is a notable decrease in the amount of light transferred into the root canal as the depth increases. This has been demonstrated to fall to levels that are inadequate for accomplishing polymerization, particularly in the apical third [1].

It is well know that the ethanol wet bonding technique works well when used on coronal dentin [39,40,41]. The results of the present study showed that ethanol, when used as a pre-treatment before adhesive system application, led to a significant increase in bond strength, above all when the 30 s etching time was tested. Bitter et al. showed results contrary to those obtained here, observing a reduction in bond strength when a multi-mode adhesive was applied in the etch-and-rinse mode after ethanol pre-treatment [42]. However, these discrepancies could be related to the different multi-mode adhesive employed. Clearfil Universal Bond Quick is developed with a cocktail of hydrophilic amide monomers which, in conjunction with an ultra-mild pH, could explain an increased bond strength when ethanol is applied over dentin. Modern etch-and-rinse adhesives that have hydrophilic properties form stronger bonds with dentin. However, they also have the downside of leaving behind higher levels of water after the evaporation of the solvent. This can lead to increased porosity in the matrix and inadequate penetration of hydrophobic monomers. The primary objective of using the ethanol wet bonding technique was to replace and reduce the water content in acid-etched dentin with ethanol. This process helps create an environment where the collagen fibers are surrounded by ethanol instead of water, enabling hydrophobic resins to penetrate the substrate effectively [43]. The use of ethanol has the potential to regulate moisture levels within the root canal by increasing the hydrophobicity of the collagen matrix. This is achieved by replacing water with ethanol [44]. In addition, significantly less micropermeability in hybrid layers has been observed for ethanol wet bonding compared to water wet bonding [45].

Pre-treatment with ethanol can extend the duration of the resin–dentin bond, enhancing the infiltration of the resin monomer and improving the formation of the hybrid layer. Ethanol, when compared to water, has superior solvent capabilities and a reduced hydrogen bonding capacity of collagen fibrils. This results in the chemical dehydration of the demineralized fibrils, generating a relatively hydrophobic matrix that reduces the hydrolysis of the interface related to water extraction from the substrate [46].

In situ zymography was utilized to scrutinize the inherent protease activities of the hybrid layer produced in radicular dentin with a multi-mode adhesive in the etch-and-rinse mode. In situ zymography [47] is a reliable and quantifiable method for comparing the relative degradation capability of resin–dentin interfaces without depending on the real degradation of the resin-sparse, water-rich denuded collagen fibrils [24]. In situ zymography quantification analysis showed that the activation of MMP gelatinolytic activity occurred in all tested groups, regardless of etching time and ethanol pre-treatment. In fact, dentin conditioning exposes collagen fibers and activates MMPs [10]. Indeed, there was a significant increase in MMP activity when the dentin was etched for 30 s, which supports previous studies that found higher enzymatic activity in over-etched dentin. Interestingly, the use of ethanol pre-treatment resulted in a significant reduction in MMP activity when etching was performed for 30 s. This suggests that ethanol pre-treatment could potentially improve the stability of the resin–dentin bond when used as a pre-treatment before adhesive application.

Ethanol has an inhibitory effect on MMPs [48]. One reason for this inhibitory effect is the ability of alcohol to create a covalent linkage between the zinc catalytic site of MMPs and the hydroxyl oxygen atom of alcohols. This interaction results in the deactivation of MMPs, enhancing the longevity of the resin–dentin bond [19].

The presence of water inside the dentinal substrate has always been considered a problem, particularly when it comes to root dentin. This is because water can trigger the breakdown of ester bonds in methacrylate materials through chemical hydrolysis, resulting in the deterioration of the hybrid layer.

There are two aspects to consider within the adhesive interface: the first is the degradation that occurs in the adhesive layer above the hybrid layers, while the second is the intrinsic collagen degradation caused by MMPs, which originate from beneath the adhesive layers over time [27]. It has previously been documented that MMPs and cysteine cathepsins degrade exposed collagen at the adhesive interface in root canal dentin [17,49]. It appears that they have a role in the gradual breakdown of collagen fibrils within the hybrid layer, which is essential for attaching resin composites to the mineralized dentin. When adhesive resins are able to effectively seal the dentin matrix and prevent water penetration, they may offer protection against collagen degradation caused by MMPs originating from the host [25,45].

However, it should be highlighted that the present study has some limitations, above all related to the study itself. It is well known that in vitro research could produce some data or results that are not as scientifically relevant as in vivo studies. Moreover, the literature review showed several studies about the collagenolytic activity of MMPs impacting bond strength stability overtime, but no clinical evidence through prospective clinical studies or metanalyses has been published in this regard. It should also be considered that there are considerable variations in the design of the push-out test; this may have an impact on the measured push-out bond strength [50].

## 5. Conclusions

Within the limitations of the present in vitro study, the following conclusions can be made:

- The ethanol wet bonding technique did not affect radicular bond strength at baseline for coronal and apical dentin; however, bonding in the apical region was shown to be less effective than in the coronal one;
- Etching time plays an important role in increasing immediate bond strength, irrespective of the use of ethanol. However, extended etching time activates a higher amount of MMPs, possibly leading to faster degradation of the hybrid layer over time.

Longer observation times are needed to validate these in vitro results and better clarify the role of extended etching time on radicular dentin adhesion.

## Figures and Tables

**Table 1:** Means and standard deviations (expressed in MPa) of push-out bond strength obtained in the different groups. Differences are considered significant at *p* < 0.05. Groups with the same superscript letters do not demonstrate a significant statistical difference (*p* > 0.05).

	Group 1	Group 2	Group 3	Group 4
coronal	10.35 ^a^ ± 3.64	13.86 ^b^ ± 3.60	18.91 ^b^ ± 4.34	21.47 ^b^ ± 6.94
apical	6.92 ^a^ ± 1.99	14.68 ^b^ ± 2.83	11.39 ^b^ ± 3.49	12.24 ^b^ ± 4.15

## Data Availability

Data supporting reported results are not provided due to ethical restrictions, but can be provided upon request.
